# Social determinants of health screening and interventions in neonatal care pathways (NICU to follow-up): a scoping review

**DOI:** 10.1007/s00431-026-06957-9

**Published:** 2026-04-20

**Authors:** Behrouz Nezafat Maldonado, Katie Evans, Martha Jones, Jessica Park, Elaine Wood, Hammad Khan, Paul Cawley

**Affiliations:** 1 Neonatal Intensive Care Unit, Evelina Children’s Hospital London, St Thomas’ Hospital, 6th Floor North Wing, Westminster Bridge Road, London, SE1 7EH UK; 2 Neonatal Medicine, Faculty of Medicine, School of Public Health, Imperial College London, Chelsea and Westminster Hospital, Chelsea and Westminster Campus, London, UK; 3 Centre for Paediatrics and Child Health, Imperial College London, London, UK

**Keywords:** Neonatal care, NICU, SDOH

## Abstract

Social determinants of health (SDOH) influence families’ ability to engage with neonatal care and may contribute to inequities in outcomes. Whilst the neonatal period presents a unique opportunity to address SDOH, it is unclear how screening and related responses are implemented across the neonatal care pathway. This review aimed to (i)o map and characterise existing screening tools and interventions used in the neonatal period to address SDOH and (ii) identify gaps in effectiveness and equity impacts. We conducted a scoping review (PRISMA-ScR) of studies from high-income countries reporting SDOH screening tools and/or interventions delivered in neonatal inpatient settings, discharge/postnatal pathways, neonatal follow-up services, or early infancy primary care. We report setting, tool content and delivery, downstream actions (referral/navigation), outcomes reported, and whether measurement properties were reported. Eight studies were included (predominantly from the USA/Canada) from 3764 studies screened. Most evidence related to implementation of screening and referral workflows rather than evaluation of downstream clinical outcomes. Screening approaches identified high levels of reported social need and commonly triggered social work involvement and referrals; only one study reported a defined “connection with resources” outcome beyond referral. Two studies evaluated equity-relevant interventions beyond screening: a digital participation intervention to support attendance at family-centred rounds and a follow-up intervention addressing structural barriers through coordinated support. Across included neonatal applications, psychometric properties of instruments were not reported, and tools were commonly adapted from other settings or locally developed. Outcomes were heterogeneous and most frequently process-focused (uptake, referrals), limiting comparability.

*Conclusion*: Evidence describing SDOH screening and responses across neonatal care pathways remains limited and largely descriptive. Future work should define a core set of SDOH domains with context-specific modules, use standardised outcome reporting, and apply a minimum reporting set for neonatal SDOH screening/intervention studies.

**Table:** 

**What is Known:** • *Studies mainly report screening/referral processes; neonatal-context psychometric evidence and standardised downstream outcomes are lacking.*	
**What is New:** • *We propose a minimum reporting set to improve how future neonatal SDOH screening/intervention studies are reported and evaluated.*

**Supplementary Information:**

The online version contains supplementary material available at 10.1007/s00431-026-06957-9.

## Introduction

Families of babies admitted to the neonatal unit are more likely to be from socio-economically disadvantaged groups [1]. Barriers such as travel costs, time away from work, childcare responsibilities, language needs, and insecure housing or food access may reduce parental presence and participation at the cot-side and during ward rounds. Lack of parent participation in their baby’s care can impact health outcomes and development [2, 3].

Paediatric organisations, including the American Academy of Pediatrics and the United Kingdom Royal College of Paediatrics and Child Health, recommend social needs screening in healthcare settings [4, 5]. Social screening has been more commonly carried out in paediatric settings compared to neonatal units. A US survey of NICUs found under a quarter of surveyed units reported standardised social determinants of health (SDOH) screening [6]. However, neonatal settings differ from general paediatrics as admissions are frequently unplanned, infants may stay for prolonged periods, and parents may face substantial logistical and emotional burdens. The neonatal admission therefore represents a potential opportunity to identify and address social risks using trauma-informed, family-centred approaches, embedded within routine care and discharge planning.

Household income, language, and access to food or housing security can all impact on families during their neonatal stay [7–9]. Parents with additional stress outside of the NICU may not engage with NICU activities compared to those in a more privileged position. This may impact skin-to-skin times, breastfeeding rates, or parental presence during the ward round [10]. Family-centred care (FCC) principles are recognised as a key ethos of neonatal care. However, FCC requires parents to be present regularly at the cot-side to work together with healthcare professionals to care for their baby [11, 12].

Previous studies in paediatrics have found that “screen and refer” programmes are feasible and acceptable [13]. Universal screening can be a powerful tool to identify families with social needs and mitigate biases and assumptions around who needs help. Despite growing interest, it remains unclear what social determinants of health (SDOH) domains are assessed in neonatal settings, how screening is carried out (who delivers it, when, and how results are acted upon), and what outcomes are reported. In addition, while screening tools are being implemented, there is limited evaluation on their psychometric properties (e.g. reliability, validity).

We carried out a scoping review to map the available tools and interventions in the neonatal period and report their characteristics and to identify key gaps to inform practice and future research.

## Methods

This scoping review was conducted in accordance with the Preferred Reporting Items for Scoping reviews (PRISMA-SCR) [14]. We performed a comprehensive electronic search using the following databases: MEDLINE, Embase, CINAHL, Web of Science, and PsychINFO. Gray literature was searched through Google Scholar and through consultation with topic experts. The search strategy combined terms for (1) social determinants/social needs/social risk, (2) neonatal/NICU/postnatal settings, and (3) screening tools/interventions/referral. The full search strategy for one database is provided in the Supplementary File.

### Aims

This review aims to:

1. Map and characterise existing screening tools and interventions used in the neonatal period to address SDOH
2. Identify gaps in reported outcomes, effectiveness evidence, and equity impacts
3. Recommend how future tools should be developed, reported, and evaluated

### Selection criteria

We used the Population-Concept-Context (PCC) framework to define the inclusion criteria. We include studies of families/caregivers of infants in the neonatal or early postnatal period (infants aged 0–12 months). We include (i) SDOH screening tools used to identify social risks/needs and report screening results and/or (ii) interventions designed to address social risks/needs (e.g. referral/navigation programmes, medico-legal support, digitally enabled family-centred care) where social factors were explicitly measured or targeted. These tools and interventions must be carried out in neonatal inpatient settings (NICU), postnatal/discharge pathways, neonatal follow-up/high-risk infant clinics, and early postnatal primary care settings (e.g. newborn or early well-child visits) to support families in the first year of life.

We included primary research studies in any language. We restricted inclusion to high-income countries as defined by the World Bank to increase comparability of health and social care systems and the transferability of service delivery models across similar resource contexts [15].

We excluded studies that did not include any SDOH screening or explicit evaluation of social risk/need and studies focused solely on universal clinical interventions not linked to social risk identification (e.g. breastfeeding initiatives delivered to all families without SDOH measurement).

We defined “interventions” as approaches intended to address barriers or needs beyond identification (e.g. navigation/support programmes or digital participation strategies), rather than screening implementation alone.

### Data extraction

We used Covidence, a web-based collaboration software platform that streamlines the production of scoping reviews and manages the screening and selection of studies [16]. Two reviewers (BNM, KE) screened titles and abstracts independently; disagreements were discussed with a third reviewer. This same review process was applied for full-text review. All authors reviewed the included studies at the end of the screening phase to confirm all studies met the inclusion criteria. We reviewed the reference lists of included studies to identify any additional studies for inclusion.

Data extraction followed a pre-defined collection form. The data extraction form was used to extract data on study characteristics including the following: author/year/country, study design, setting, participant characteristics, description of the screening tool/intervention, delivery mode and staff involved, training requirements, languages and health-literacy adaptations, SDOH domains assessed, referral pathways/resources, and reported outcomes (e.g. screening uptake, positive screens, referrals, access to benefits, parent-reported outcomes). Data extraction was carried out by a primary reviewer and confirmed by a second reviewer to ensure that accurate information had been recorded. Any discrepancies were resolved through discussion between the reviewers.

### Synthesis

We conducted descriptive mapping and narrative synthesis. Due to heterogeneity in study designs, tools, outcomes, and settings, meta-analysis was not planned. Findings are presented as (i) characteristics of screening tools and interventions, (ii) SDOH domains assessed, and (iii) outcomes reported.

### Critical quality appraisal

Critical appraisal is not required for scoping reviews.

### Patient and public involvement

No patients or members of the public were involved in this scoping review. As this was a review of available literature, no ethics approval was necessary. The wider project looking at social determinants of health in neonatal care has been registered with the local research and development office.

### Ethics

As this study synthesised available literature, ethics approval was not required.

## Results

### Study selection

**Fig. 1:**
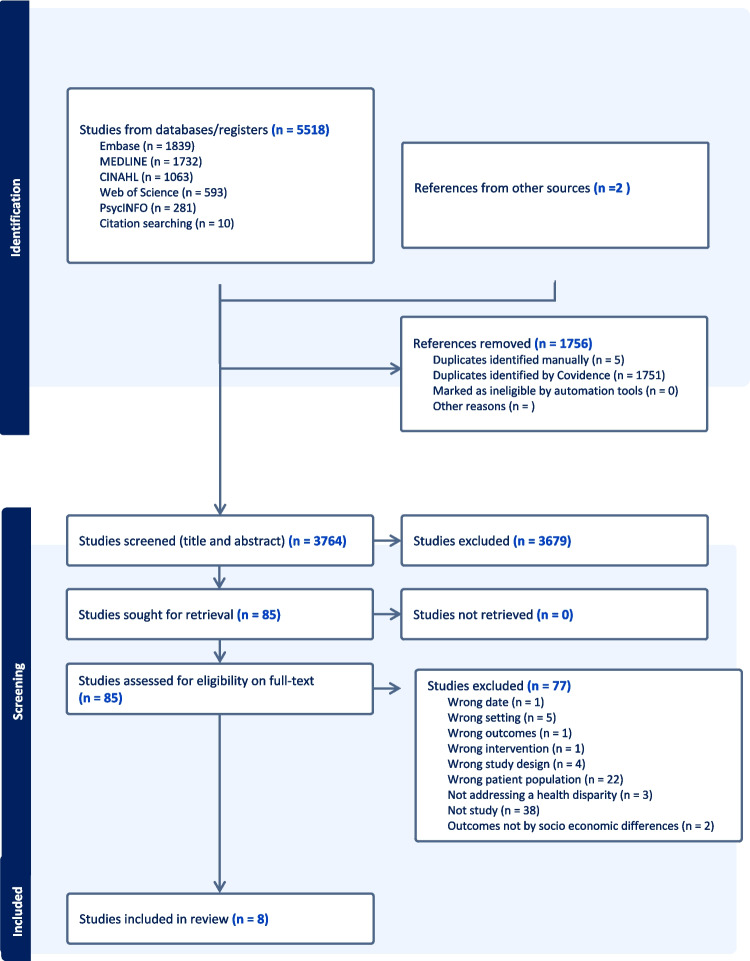
PRISMA flow diagram

Eight studies were included following full-text review of 3764 unique titles and abstracts. Figure 1 shows the PRISMA flow diagram. Reasons for exclusion of 77 studies following full-text review are listed in the Supplementary File. This reflects that neonatal SDOH evidence is dominated by descriptive studies that focus on measures of disparities or their associations, with few studies reporting on a screening approach or intervention that can be evaluated.

### Study characteristics

All included studies were conducted in high-income settings, predominantly in the USA and Canada, and most were single-centre evaluations [17–24]. Of the eight included studies, four described screening tools and workflows to identify social risks/needs during the inpatient admission, and four focused on discharge/postpartum and follow-up settings. Study designs were largely descriptive, including quality improvement (QI) reports, observational cohort analyses, and cross-sectional surveys; one study was a post-hoc subgroup analysis derived from a pilot randomised trial. Studies spanned the neonatal care pathway (inpatient, discharge/postnatal care, community settings, and neonatal follow-up) and addressed access to resources, medico-legal support, and technology-enabled engagement. Study characteristics are summarised in Tables 1 and 2. All included studies were published within the last 6 years, reflecting recent interest in this area; despite no language restrictions, all were published in English.

**Table 1:** Screening and inpatient interventions (identification + referral)

Study	Country	Design	Setting/population	Screening/intervention	Outcomes reported	Key findings relevant to inequalities/SDOH
Lagatta (2024)	USA	Prospective cohort (embedded in parent experience study)	Level IV NICU; parents of infants in NICU	PRAPARE tablet-based SDOH screening with chart review of social work follow-up	% with ≥ 1 need; referral actions; associations with infant illness; post-discharge utilisation (ED)	19% (61/319) reported ≥ 1 resource need; > half of positive screens resulted in new resource referrals; infants whose parents’ reported needs were more likely extremely preterm/higher acuity/longer LOS; after discharge more likely ED use
Cordova-Ramos (2023)	USA	QI report	NICU; “eligible families” (longer stay/criteria)	Systematic SDOH screening (THRIVE) + referral connection workflow	Screening uptake; needs detected; connections to resources	Reported increased systematic screening and increased recognition of unmet social needs with an implemented referral pathway
Travia (2023)	USA	QI report	71-bed level IV NICU; families of admitted neonates	Food insecurity screening (2-item FI questions) + referral process	Screening rate; referral after positive screen	Aim achieved: 95% screening rate within 6 months; those screening positive were referred for additional resources
Rosenthal (2024)	USA	Post-hoc subgroup analysis of RCT	NICU; parents/guardians of enrolled infants	Virtual FCR option (Zoom) vs usual care	Parent FCR attendance rates; differential effects by social subgroups	Intervention arm had 3.36 × higher attendance rate (95% CI 2.66–4.23). Relative benefit was greater for racial/ethnic minorities (2.15 × better), no college education (2.68 × better), and worse neighbourhood health conditions (4.14 × better)

**Table 2:** Discharge, postpartum, and NICU follow-up interventions

Study	Country	Design	Setting/population	System/intervention	Outcomes reported	Key findings relevant to inequalities/access
Flores-Fenlon (2019)	USA	Cross-sectional	High-risk infant follow-up clinic; NICU graduates/preterm infants	Assessment of technology access (email/text/smartphone) as a potential resource for discharge transition + engagement	Parent QoL (MCQLI); enrolment in early intervention (EI); other programme enrolment	Access to email/texting/smartphone associated with higher parent QoL; adjusted differences in MCQLI included email + 8.74, texting + 16.96, smartphone + 12.01 (significant)
Redd (2018)	USA	Descriptive cohort/service evaluation	NICU graduates (0–6 years coordinated model)	Coordinated medical–legal partnership + action plan spanning health/education/social services	Parenting stress; child development; “action items” resolved; access to services	25 families: developmental/parent stress metrics improved; ~ 80% of action items resolved at follow-up
Russell (2018) (HBHC)	Canada	Cross-sectional tool evaluation	Postpartum families pre-discharge (community home visiting pathway)	HBHC screen to identify families needing home visiting services	Variables associated with high-risk classification	Factors linked with higher risk included teenage first-time parent, single parent, low education, newcomer support needs, money concerns, disability affecting parenting
Friedman (2021)	USA	Resident-led QI	Urban primary care resident/faculty practice; newborn and 1-year well visits	Parent-completed paper SDOH screening form (9 questions) + workflow changes (PDSA cycles) + resource lists ± social work referral	Complete screening rate; positive screen rate; documented interventions; provider comfort	Complete screening for all 9 questions increased 24% → 43% at newborn visits and 28% → 83% at 1-year visits; screens identifying ≥ 1 need increased 8% → 19%; documented provider response to identified need increased 20% → 40%. Practice served a largely Hispanic/immigrant, low-income community

### Inpatient screening and interventions

Four studies evaluated approaches delivered during the inpatient admission (Table 1). Three studies focused on screening for social risks/needs, reporting screening uptake, prevalence of identified needs, and downstream actions such as social work involvement and referrals [17–19]. One unit-based evaluation used the Protocol for Responding to and Assessing Patients’ Assets, Risks and Experiences (PRAPARE), a multi-domain questionnaire completed at the bedside on a digital tablet, covering demographics and resource needs (e.g. food, clothing and transportation), safety, mental health, and social support. Approximately one-fifth of screened families disclosed at least one resource need, and documented actions included referral to social work and external services. One QI study implementing systematic screening reported a high burden of social needs among families screened and increases over time in both screening uptake and a defined “connection with resources” outcome [18]. A separate QI initiative implemented routine food insecurity screening in a level IV NICU, achieving high screening coverage within 6 months, with referral processes triggered for families screening positive [19].

One study evaluated a unit-level intervention to support equitable participation in family-centred care by offering parents the option to join family-centred rounds virtually [20]. Zoom was used to enable bidirectional audio-visual participation. The study reported higher parental attendance in the intervention arm and suggested greater relative improvements among groups experiencing social disadvantage (e.g. lower educational attainment and poorer neighbourhood conditions), indicating that digital participation approaches may mitigate access barriers during inpatient care.

### Discharge and follow-up actions

Four studies evaluated SDoH screening or related supports spanning discharge/postpartum systems and follow-up (Table 2). One large Canadian postpartum programme evaluated a structured screen used to triage families to a public health in-depth assessment/home-visiting pathway and identified demographic and socioeconomic factors associated with classification as high risk [23]. Although not NICU-specific, this study illustrates how population discharge-to-community screening systems may identify needs and highlights that neonatal admissions may follow distinct downstream pathways.

Within early-life outpatient contexts, a QI study in primary care improved completion of a parent-completed SDOH screening tool at newborn and 1-year visits and increased documentation of provider responses to identified needs [24]. Two follow-up clinic studies addressed post-discharge structural barriers and access. One cross-sectional study in a high-risk infant follow-up setting identified technology access (e.g. email/texting/smartphone access) as being associated with caregiver quality of life and early intervention enrolment [21]. Another service evaluation described a coordinated medico-legal partnership and structured action planning model for NICU graduates, reporting completion of most action items and improvements in caregiver stress/hassles and child developmental measures over follow-up [22].

### SDOH domains captured across screening tools

Across included screening approaches, domains varied widely (Table 3). Housing and food insecurity were among the most commonly captured domains across inpatient screening tools and follow-up assessments. Financial strain and employment/education domains were included in several multi-domain screens, while safety and interpersonal violence were captured in some tools used outside the inpatient setting. One follow-up study explicitly assessed digital access (email/texting/smartphone) as a potential determinant of ability to engage with services and linked this to caregiver outcomes and service enrolment. Overall, the heterogeneity in domains and measurement approaches limited direct comparability of screening yield across settings.

**Table 3:** SDOH domains captured by screening tools

SDOH domain	PRAPARE (Lagatta 2024)	THRIVE (Cordova-Ramos 2023)	HBHC screen (Russell 2018)	Food insecurity screen (Travia 2023)	HelpSteps-adapted + tech access (Flores-Fenlon 2019)	Outpatient SDOH screen (Friedman 2021)
Housing instability/homelessness	✓	✓	✓		✓	
Food insecurity	✓	✓		✓	✓	
Utilities insecurity	✓	✓				
Transportation barriers	✓	✓				
Income/financial strain	✓	✓	✓		✓	
Employment	✓	✓	✓			
Education	✓	✓	✓			
Insurance/healthcare access	✓		✓		✓	
Social support/isolation	✓				✓	
Safety/interpersonal violence	✓				✓	✓
Stress/mental health	✓				✓	
Language/literacy needs	✓					
Parenting/family context			✓			✓
Environmental risks (e.g. smoke/lead)						✓
Digital access (email/text/smartphone)					✓	

Key: ✓ = domain explicitly included in the screening tool or structured assessment as used in the included study; blank = not reported/not part of the tool

### Outcomes and measures reported

Outcomes reported were predominantly process and intermediate outcomes (Table 4). Process outcomes included screening uptake/completion and implementation feasibility; intermediate outcomes included the prevalence of identified needs and documentation of social work/provider responses and referrals. Only one QI study reported a defined “connection with resources” outcome beyond referral provision [18]. Family participation outcomes (attendance at rounds) were reported for the virtual-rounds intervention, with effect estimates and equity-relevant subgroup comparisons. Caregiver outcomes were variably reported, including caregiver quality of life and parenting stress/hassles measures in follow-up settings, alongside some child developmental outcomes and early intervention enrolment measures. Overall, outcome heterogeneity and limited standardisation of “referral” and “connection” measures constrained synthesis across studies.

**Table 4:** Outcome measures across the studies included

Outcome category	What was measured	Measures/definitions used in included studies	Studies reporting
Implementation/process	Screening uptake/completion	% eligible screened; % fully completed; screening rate over time	Cordova-Ramos (uptake); Travia (screening rate); Friedman (complete screen rates)
Need prevalence/yield	Positive screen(s); number of needs	% with ≥ 1 need; count of need domains; “ ≥ 1 unmet need”	Lagatta (≥ 1 resource need); Cordova-Ramos (≥ 1 need; multiple needs); Friedman (≥ 1 need screens)
Referral/response	Actions after a positive screen	Social work encounter; new resource referrals; documented provider response	Lagatta (repeat SW; new referrals); Cordova-Ramos (referrals provided); Friedman (documented provider response)
Resource connection	Whether families actually connected with resources	“Connection with resources” metric (as defined by study)	Cordova-Ramos (connection improved)
Care participation/engagement	Participation in family-centred care processes	Parent attendance rate at rounds; rate ratios/relative increases	Rosenthal (attendance rate ratio)
Caregiver outcomes	QoL, stress, parenting hassles	MCQLI caregiver QoL; Parenting Hassles Scale/stress measures	Flores-Fenlon (MCQLI); Redd (parenting hassles)
Infant/child outcomes	Development/functional outcomes; programme enrolment	WIDEA-FS; Bayley; EI enrolment; school/IEP-related outcomes	Redd (WIDEA-FS/Bayley; action plan outputs); Flores-Fenlon (EI enrolment associations)
Equity/subgroup effects	Differential effects/uptake by social group	Comparisons by race/ethnicity/language/education/insurance/neighbourhood	Cordova-Ramos (screening uptake vs race/language); Rosenthal (subgroup-modified effects)
Psychometrics	Reliability/validity of tools	Reliability/validity testing or formal psychometric evaluation	Not reported within included screening evaluations (tools used rather than validated in neonatal settings)

## Discussion

### Principal findings

This scoping review mapped SDOH-related screening approaches and a small number of interventions across the neonatal care pathway, spanning inpatient care, discharge/postpartum systems, and follow-up. The evidence base was recent and geographically concentrated in North America and was dominated by descriptive implementation studies. Across inpatient settings, screening approaches identified families reporting social risks/needs and commonly prompted downstream actions such as social work involvement and referrals; however, outcomes were largely process-focused and only one study reported a measure beyond referral provision (a defined “connection with resources” outcome). Beyond screening, the review identified two equity-relevant interventions with outcomes reported: a digital participation intervention to increase parental attendance at family-centred rounds, and a follow-up service model incorporating coordinated medico-legal (legal aid) support and structured action planning for NICU graduates, which reported high completion of action items alongside improvements in caregiver stress/hassles and child developmental measures over follow-up. Overall, the literature provides early examples of how neonatal services can operationalise social risk identification and response, but remains limited in standardised outcomes, longer-term impacts, and reporting of measurement properties in neonatal contexts.

### Interpretation and clinical relevance

The findings suggest that the neonatal admission and early postnatal period represent opportunities to identify and respond to social risks/needs that may constrain families’ ability to engage with care. Many neonatal services already provide some level of social work support with the studies identified here providing ways to make this support more systematic through structured screening and defined workflows for follow-up and referral. From a clinical perspective, the most transferable learning relates to implementation, particularly around who administers screening, when it is offered, how results are recorded, and how actions are triggered and tracked. Importantly, the current evidence does not allow firm conclusions about the effects of screening on infant clinical outcomes, but it does highlight that families report substantial unmet needs, and that services can integrate screening with referral processes.

The limited number of intervention studies also points to promising directions aligned with everyday neonatal practice. The virtual participation intervention targets an immediate mechanism through which social circumstances may influence care, impacting on parental ability to attend rounds and participate in decision-making. However, placing emphasis on digital health interventions could inadvertently widen health inequalities. Digital health literacy is increasingly recognised as an important determinant of health, and for families with low socioeconomic status, limited digital literacy and reduced access to technology may create barriers to engagement [25]. Therefore, implementing digital or virtual interventions should be accompanied by structural measures to support equitable access, such as provision of appropriate connectivity and technology and education to build digital literacy. Meanwhile, follow-up interventions such as medico-legal partnerships address structural barriers beyond the inpatient setting that can affect access to benefits, early intervention, and educational supports. Together, these approaches emphasise that reducing inequities in neonatal outcomes is likely to require systematic recognition of social risks/needs and interventions that directly reduce barriers to engagement and service access during transitions from hospital to community care.

### Screening tools and domains

Tools varied substantially in scope and content, ranging from brief targeted screening (e.g. food insecurity) to multi-domain questionnaires including housing, financial strain, transport, safety, and mental health. Such variation is unsurprising given differences in population needs and local service configuration. However, heterogeneity in domains and definitions reduces comparability across settings and complicates interpretation of screening “yield”. A pragmatic approach for neonatal services and future research would be to co-develop a core set of domains relevant across neonatal pathways (e.g. housing and food insecurity, financial strain, transport barriers, language needs), with the option to add context-specific modules where relevant. This would balance local relevance with cross-site comparability.

A key gap is that included studies did not report neonatal-context psychometric properties for the instruments used. Some instruments may have established measurement evidence in other populations, but their performance in neonatal contexts—across languages, cultures, and modes of administration—remains unclear from the included literature. Future work should explicitly report instrument versions and adaptations and evaluate validity, reliability, and measurement equivalence in neonatal settings where screening is intended to inform care and resource allocation. We have summarised suggestions for future work in Table 5.

**Table 5:** Evidence gaps identified to guide future research

Area	What the current literature provides (from included studies)	Key evidence gaps	Minimum reporting set for future studies
Population and setting definition	Mostly single-centre NICU/QI studies and follow-up clinic evaluations; some postpartum population programmes	Limited multi-site evidence; inconsistent description of NICU level, catchment, and discharge pathways	NICU level/type; country/health system; recruitment window; inclusion/exclusion (e.g. LOS threshold); denominators for “eligible” vs “screened”; discharge/follow-up pathway description
Timing and delivery of screening	Screening sometimes embedded during admission (tablet or staff-led), sometimes in outpatient well visits; timing varies	Unclear optimal timing (admission vs pre-discharge vs repeated); limited reporting of staff time/training burden	Timepoint(s) (admission, weekly, pre-discharge, follow-up); who administers; mode (paper/tablet/EHR/verbal); average time to complete; staff training content/duration; completion supports (interpreters, literacy aids)
Domains captured (content validity)	Wide variation in domains (housing/food/transport common); digital access appears in follow-up	No consensus “core” neonatal domain set; limited attention to language/health literacy and immigration-related barriers	Domains mapped to a framework (e.g. PROGRESS-PLUS); rationale for domain selection; languages offered; reading level; caregiver burden/acceptability; data privacy/confidentiality approach
Psychometrics and measurement properties	Tools are used (PRAPARE, THRIVE adaptations), but neonatal-specific psychometrics not reported	Reliability/validity/responsiveness not tested in neonatal contexts; uncertain measurement equivalence across languages/cultures	Instrument name/version; any adaptations; evidence of validity/reliability in target setting; plan for psychometric evaluation
Screening uptake and yield	Studies often report uptake and “positive screens”, sometimes with high unmet need burden	Variable denominators and definitions of “positive”; lack of standard metrics across studies	Denominators: eligible, approached, screened, complete; definition of positive screen; prevalence per domain; number of needs per family; missing data per item/domain
Referral processes and response fidelity	Some quantify referrals and social work follow-up (e.g. new referrals after PRAPARE; provider response documentation)	Poorly standardised definitions (“referral”, “resource provided”, “navigation”); limited reporting of fidelity/implementation	Definitions of: referral offered, referral accepted, follow-up; response time; staff role responsible for each step
Access to resources (beyond referral)	One NICU QI study reports “connection with resources” as an outcome	Rarely measured; no common definition; little long-term follow-through	Define timeframe for assessment (e.g. pre-discharge, 30/90 days); method (EHR, phone follow-up, registry)
Family-centred care participation/engagement	Virtual rounds intervention reports attendance effect sizes and equity-relevant subgroup effects	Few trials; limited measurement of parental participation (rounds, skin-to-skin, caregiving)	Participation outcomes (attendance to wardround, bedside time, involvement in care decisions); effect sizes (RR/IRR/OR with CI); implementation costs and staffing
Caregiver outcomes	Follow-up studies report caregiver QoL and parenting stress/hassles	Inconsistent measures; limited trauma-informed acceptability and potential harms reporting	Pre-specified caregiver outcomes (QoL, stress, empowerment); validated instruments; acceptability/comfort; adverse effects (distress, mistrust), and mitigation plan
Infant/child outcomes	Some follow-up interventions report developmental outcomes and service enrolment (EI)	Sparse evidence linking screening/interventions to infant health or developmental outcomes; limited longitudinal follow-up	Infant/child outcomes (development, readmissions/ED use, growth where relevant); timeframe; linkage methods; confounder adjustment strategy for observational analyses
Equity and subgroup reporting (PROGRESS-PLUS)	A minority report subgroup analyses or check screening uptake by race/language	Limited reporting of differential benefit/harm; limited intersectional analyses	A priori equity variables (race/ethnicity, language, education, insurance, neighbourhood deprivation, migration); stratified uptake/yield/referral/connection; differential effects (interaction terms)
Implementation determinants and costs	Some QI work describes workflow and barriers qualitatively	Rare use of implementation frameworks; little cost/time/capacity reporting	Implementation framework (e.g. RE-AIM/CFIR); barriers/facilitators; staff time; training costs; sustainability plan; scalability considerations
Study design and rigour	Mix of QI evaluations, cohort analyses, and a trial subgroup analysis	Few controlled designs; limited causal inference; inconsistent reporting standards	Pre-specified primary outcomes; comparator/usual care where feasible; analytic plan; handling missingness; sensitivity analyses; protocol registration

### Implications for neonatal practice and research

**Fig. 2:**
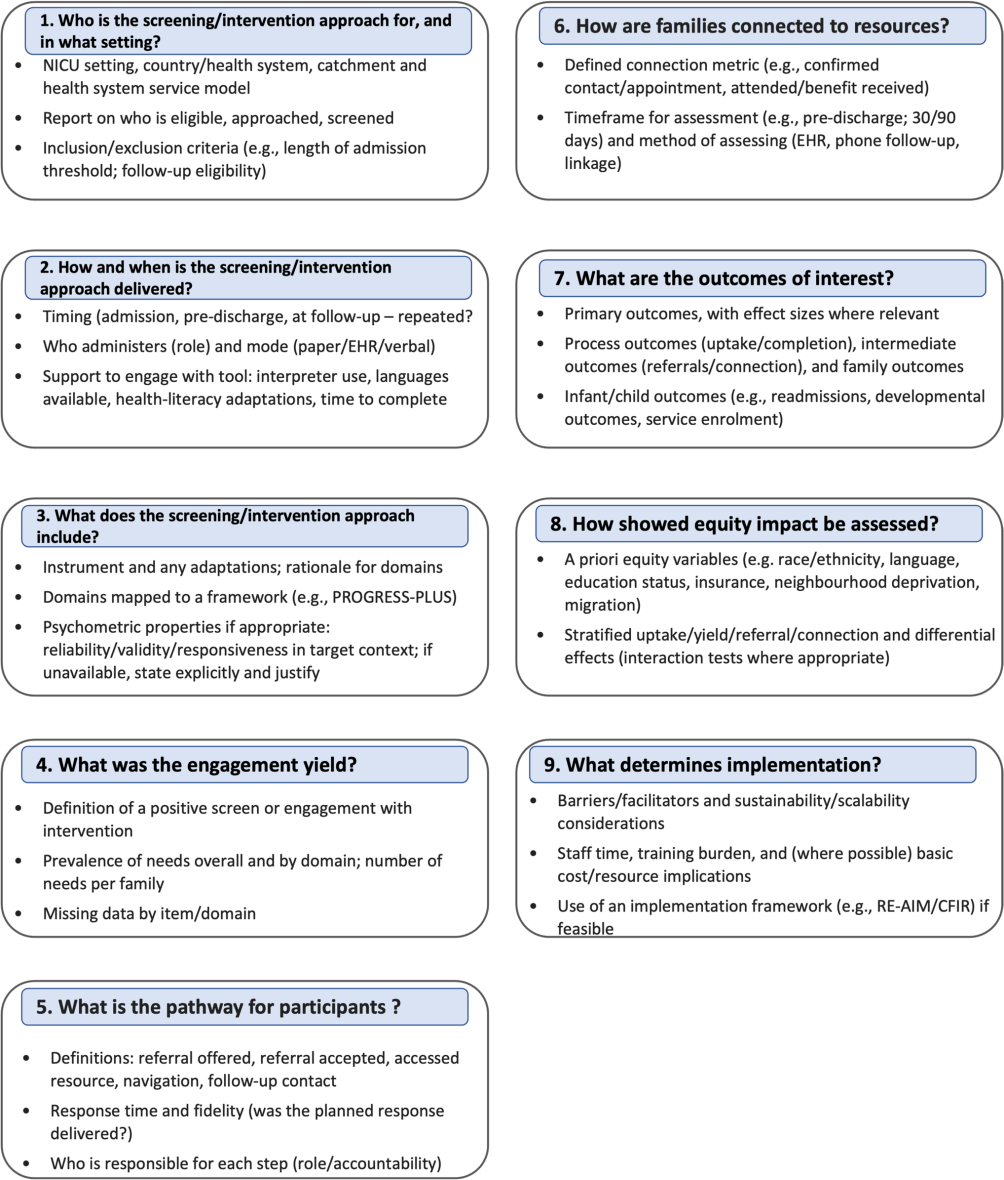
Recommended reporting questions for studies of social determinants of health (SDOH) screening and related interventions across neonatal inpatient, discharge/postnatal, and follow-up settings. In this figure, “screening/intervention approach” includes screening questionnaires/tools, referral or navigation workflows, and interventions addressing social needs (e.g. social work linkage, community/charity support, food bank referral, or medico-legal support)

Despite limited intervention evidence, the mapped studies support several practical considerations for neonatal services considering SDOH screening and response. Screening should be embedded within a defined workflow with clear accountability for action (review, referral, follow-up), and services should measure “connection to resources”, not referral alone. Addressing barriers to participation in care such as digital inclusion may offer a direct route to improving family-centred care for socially disadvantaged families. Delivery should be trauma-informed and equity-focused, including language support, literacy-appropriate materials, and attention to confidentiality and cultural safety. To support comparability and implementation fidelity, we propose a minimum reporting set (Fig. 2).

Future research should prioritise neonatal-context measurement evidence for tools used (including psychometrics and measurement equivalence), development of a core domain set with context-specific modules, and evaluation using rigorous designs (e.g. pragmatic trials, interrupted time series, stepped-wedge, or controlled before-after studies) (Table 5). Studies should report standardised outcomes spanning process, intermediate, and family/infant outcomes, and include a priori equity analyses (e.g. PROGRESS-PLUS variables) to test differential effects and avoid widening disparities. Implementation science frameworks may support assessment of feasibility, costs, sustainability, and unintended consequences.

### Strengths and limitations

This review maps SDOH screening and related interventions across the neonatal pathway rather than focusing solely on inpatient screening tools and provides practice-facing outputs (Fig. 2) to support service implementation and future research. Limitations include the small number of intervention evaluations, geographic concentration of included studies, heterogeneity in tools and outcomes, and predominance of descriptive designs, which limits inference about downstream clinical impact.

## Conclusions

Evidence describing SDOH screening and responses across neonatal care pathways is emerging but remains limited and largely descriptive. Screening approaches identify substantial unmet social needs and commonly prompt referrals, but neonatal-context measurement evidence and standardised downstream outcomes are scarce. Future work should define core domains, evaluate instrument performance in neonatal contexts, and test interventions that reduce structural barriers to engagement and resource access, using rigorous designs and equity-focused reporting.

## Supplementary Information

Below is the link to the electronic supplementary material.

ESM 1 Supplementary Material 1 (DOCX 29.6 KB)

## Data Availability

No datasets were generated or analysed during the current study.

## References

[1] Adappa R, Barr S (2023) Social determinants of health and the neonate in the neonatal intensive care. Paediatr Child Health 33(6):154–157. 10.1016/j.paed.2023.03.002

[2] Nist MD, Robinson A, Pickler RH (2023) Parental participation in preterm infant feeding in the neonatal intensive care unit. MCN Am J Matern Child Nurs 48(2):76–81. 10.1097/NMC.0000000000000890 36472494 10.1097/NMC.0000000000000890 PMC9974565

[3] Pineda R, Bender J, Hall B, Shabosky L, Annecca A, Smith J (2018) Parent participation in the neonatal intensive care unit: predictors and relationships to neurobehavior and developmental outcomes. Early Hum Dev 117:32–38. 10.1016/j.earlhumdev.2017.12.008 29275070 10.1016/j.earlhumdev.2017.12.008 PMC5856604

[4] Poverty and Child Health (2024). Available from: https://www.aap.org/en/patient-care/poverty-and-child-health/srsltid=AfmBOopB3hjLI9FnJJpOPoXVywDzs4qnkz1SxiWKscpxeIPTbRyxX9NV. Accessed 16 Dec 2024

[5] RCPCH (2024) Spot the difference - addressing social determinants in everyday management. Available from: https://www.rcpch.ac.uk/news-events/news/spot-difference-addressing-social-determinants-everyday-management. Accessed 16 Dec 2024

[6] Cordova-Ramos EG, Kerr S, Heeren T, Drainoni ML, Garg A, Parker MG (2022) National prevalence of social determinants of health screening among US neonatal care units. Hosp Pediatr 12(12):1040–1047. 10.1542/hpeds.2022-006767 36317484 10.1542/hpeds.2022-006767 PMC9814031

[7] Bourque SL, Weikel BW, Palau MA, Greenfield JC, Hall A, Klawetter S et al (2021) The association of social factors and time spent in the NICU for mothers of very preterm infants. Hosp Pediatr 11(9):988–96. 10.1542/hpeds.2021-005861 34426486 10.1542/hpeds.2021-005861 PMC10037762

[8] Lakshmanan A, Song AY, Belfort MB, Yieh L, Dukhovny D, Friedlich PS et al (2022) The financial burden experienced by families of preterm infants after NICU discharge. J Perinatol 42(2):223–30. 10.1038/s41372-021-01213-4 34561556 10.1038/s41372-021-01213-4 PMC8460846

[9] McCarty DB, Golden SD, Ferrari RM, Zvara BJ, Wilson WD, Shanahan ME (2024) Sociodemographic characteristics of maternal presence in neonatal intensive care: an intersectional analysis. J Perinatol. 10.1038/s41372-024-02175-z 10.1038/s41372-024-02175-z 39572691

[10] Lazzerini M, Gomez DBC do A, Azzimonti G, Bua J, Neto WB, Brasili L et al (2024) Parental stress, depression, anxiety and participation to care in neonatal intensive care units: results of a prospective study in Italy, Brazil and Tanzania. bmjpo 8(Suppl 2). 10.1136/bmjpo-2024-002539 10.1136/bmjpo-2024-002539 PMC11664376 39106992

[11] Hutchfield K (1999) Family-centred care: a concept analysis. J Adv Nurs 29(5):1178–1187. 10.1046/j.1365-2648.1999.00987.x 10320502 10.1046/j.1365-2648.1999.00987.x

[12] Toivonen M, Lehtonen L, Löyttyniemi E, Ahlqvist-Björkroth S, Axelin A (2020) Close collaboration with parents intervention improves family-centered care in different neonatal unit contexts: a pre–post study. Pediatr Res 88(3):421–428. 10.1038/s41390-020-0934-2 32380505 10.1038/s41390-020-0934-2 PMC7478938

[13] Part I: A quantitative study of social risk screening acceptability in patients and caregivers - ScienceDirect (2024) Available from: https://www-sciencedirect-com.iclibezp1.cc.ic.ac.uk/science/article/pii/S0749379719303186. Accessed 16 Dec 2024

[14] Tricco AC, Lillie E, Zarin W, O’Brien KK, Colquhoun H, Levac D et al (2018) PRISMA extension for scoping reviews (PRISMA-ScR): checklist and explanation. Ann Intern Med 169(7):467–73. 10.7326/M18-0850 30178033 10.7326/M18-0850

[15] World Bank Group (2025) Understanding country income: World Bank Group income classifications for FY26. World Bank Blogs. https://blogs.worldbank.org/en/opendata/understanding-country-income--world-bank-group-income-classifica. Accessed 6 Jan 2026

[16] Covidence systematic review software, Veritas Health Innovation, Melbourne, Australia. Available at https://www.covidence.org

[17] Lagatta J, Hoffman C, Harris M, Acharya K, Malnory M, Cohen S (2024) Impact of systematic screening for social determinants of health in a level IV neonatal intensive care unit. Res Square (101768035). 10.21203/rs.3.rs-4656439/v1 10.1038/s41372-024-02096-x 39244612

[18] Cordova-Ramos EG, Jain C, Torrice V, McGean M, Buitron de la Vega P, Burke J et al (2023) Implementing social risk screening and referral to resources in the NICU. Pediatrics. 10.1542/peds.2022-058975 36919445 10.1542/peds.2022-058975 PMC10797529

[19] Travia K, Kohler. JA Sr, Akpan US (2023) Implementing food insecurity screening in a level IV neonatal intensive care unit. J Perinatol 43(9):1183–8. 10.1038/s41372-023-01709-1 10.1038/s41372-023-01709-1 37400495

[20] Rosenthal J, Hoffman K, Sauers-Ford H, Stein D, Haynes S, Tancredi D (2024) Differential impact of virtual family-centered rounds in the neonatal intensive care unit by social factors: a post hoc subgroup analysis. Telemed E-Health. 10.1089/tmj.2024.0176 10.1089/tmj.2024.0176 PMC11698681 39119710

[21] Flores-Fenlon N, Song AY, Yeh A, Gateau K, Vanderbilt DL, Kipke M et al (2019) Smartphones and text messaging are associated with higher parent quality of life scores and enrollment in early intervention after NICU discharge. Clin Pediatr (Phila) 58(8):903–11. 10.1177/0009922819848080 31088122 10.1177/0009922819848080 PMC8362840

[22] Redd L, Belcher R, Dotts B, Andrews B (2018) A silver lining for neonatal intensive care (NICU) graduates: coordinated services from 0–6 years. International Public Health Journal 10(4):325–332

[23] Russell K, Gilbert L, Hebert D, Ali A, Taylor RSL, Hendriks A (2018) Ontario’s healthy babies healthy children screen tool: identifying postpartum families in need of home visiting services in Ottawa, Canada. Can J Public Health 109(3):386–394. 10.17269/s41997-018-0052-7 29981082 10.17269/s41997-018-0052-7 PMC6964612

[24] Friedman S, Caddle S, Motelow JE, Meyer D, Lane M (2021) Improving screening for social determinants of health in a pediatric resident clinic: a quality improvement initiative. Pediatr Qual Saf 6(4):e419. 10.1097/pq9.0000000000000419 34235349 10.1097/pq9.0000000000000419 PMC8225364

[25] Estrela M, Semedo G, Roque F, Ferreira PL, Herdeiro MT (2023) Sociodemographic determinants of digital health literacy: a systematic review and meta-analysis. Int J Med Inform 1:105124. 10.1016/j.ijmedinf.2023.105124 10.1016/j.ijmedinf.2023.105124 37329766

