# Supplementary material for: Social determinants of health screening and interventions in neonatal care pathways (NICU to follow-up): a scoping review
Source: Eur J Pediatr. 2026 Apr 20;185(5):277. doi: 10.1007/s00431-026-06957-9 (PMC13092529; doi:10.1007/s00431-026-06957-9)
Supplement: Supplementary file 1 — Supplementary Material 1 (DOCX 29.6 KB) [file 431_2026_6957_MOESM1_ESM.docx]

**Supplementary Files:**

**Supplementary File 1: MEDLINE Search Strategy**

| (((("social determinants of health" OR "social risk factors" OR "social needs " OR "social drivers "OR "health disparities" OR "socioeconomic factors" OR "race" OR "ethnicity")) |
| --- |
| AND |
| (("neonate" OR "newborn" OR "preterm infant" OR "neonatal intensive care" OR "NICU" OR "infant, premature"))) |
| AND |
| (("intervention" OR "social support" OR "screening"))) |

**Supplementary File 2: Excluded on full text screening**

| **Study ID** | **Exclusion Reason** |
| --- | --- |
| Guerra 2024 | No intervention / epi descriptive study ; |
| Cordova-Ramos 2024 | No intervention / epi descriptive study ; |
| Orr 2024 | Wrong patient population; |
| LeeKing 2024 | No intervention / epi descriptive study ; |
| Schneider 2024 | No intervention / epi descriptive study ; |
| Anyigbo 2024 | Wrong patient population; |
| vandeKamp 2023 | No intervention / epi descriptive study ; |
| Malin 2023 | Outcomes not by socio economic differences; |
| Sosnowski 2023 | No intervention / epi descriptive study ; |
| Brumbaugh 2023 | No intervention / epi descriptive study ; |
| Younge 2023 | No intervention / epi descriptive study ; |
| Sullivan 2023 | Wrong patient population; |
| Fraiman 2022 | No intervention / epi descriptive study ; |
| McGowan 2022 | No intervention / epi descriptive study ; |
| Richardson 2021 | No intervention / epi descriptive study ; |
| Reichman 2021 | No intervention / epi descriptive study ; |
| Hemingway 2021 | No intervention / epi descriptive study ; |
| Gateau 2021 | No intervention / epi descriptive study ; |
| Parker 2021 | No intervention / epi descriptive study ; |
| Hajdu 2020 | No intervention / epi descriptive study ; |
| Montoya-Williams 2020 | Wrong patient population; |
| Dunn 2020 | No intervention / epi descriptive study ; |
| Hall 2020 | No intervention / epi descriptive study ; |
| Morrison 2019 | Wrong patient population; |
| Patel 2019 | No intervention / epi descriptive study ; |
| Merewood 2019 | No intervention / epi descriptive study ; |
| Pantell 2019 | Wrong patient population; |
| Forestieri 2019 | No intervention / epi descriptive study ; |
| Fowler 2018 | No intervention / epi descriptive study ; |
| Spittle 2018 | Not addressing a health disparity ; |
| Morrison 2017 | Wrong setting; |
| Kenyhercz 2017 | Wrong study design; |
| Pontoppidan 2016 | Wrong demographic ie not targeting a social risk group ; |
| Braid 2015 | No intervention / epi descriptive study ; |
| Johnson 2015 | No intervention / epi descriptive study ; |
| hIarlaithe 2014 | No intervention / epi descriptive study ; |
| McFarlane 2013 | Wrong patient population; |
| Goff 2013 | Wrong study design; |
| Pritchard 2013 | No intervention / epi descriptive study ; |
| Wakeel 2013 | No intervention / epi descriptive study ; |
| Pendleton 2012 | No intervention / epi descriptive study ; |
| McCormick 2012 | Wrong patient population; |
| Candelaria 2011 | No intervention / epi descriptive study ; |
| Hamad 2011 | Wrong intervention; |
| Meijssen 2011 | Not addressing a health disparity; |
| Twohig 2016 | No intervention / epi descriptive study ; |
| Pineda 2013 | No intervention / epi descriptive study ; |
| Preyde 2007 | Wrong date; |
| Dion 2022 | Wrong patient population; |
| M√•lqvist 2015 | Wrong patient population; |
| McConnell 2023 | Wrong patient population; |
| Gols√§ter 2024 | Wrong patient population; |
| Norr 2003 | Wrong patient population; |
| Yeary 2022 | Not addressing a health disparity ; |
| Sokol 2021 | No intervention / epi descriptive study ; |
| Higginbotham 2019 | Wrong patient population; |
| Driver 2021 | No intervention / epi descriptive study ; |
| Jani 2021 | Wrong setting; |
| Goldfeld 2024 | Wrong patient population; |
| Dmowska 2016 | Wrong patient population; |
| Pantell 2020 | Wrong patient population; |
| Acri 2015 | Wrong patient population; |
| Hannan 2013 | Wrong patient population; |
| Mekhail 2023 | Wrong patient population; |
| Blair 2020 | No intervention / epi descriptive study ; |
| Samuels-Kalow 2024 | Wrong patient population; |
| Litt 2024 | Wrong outcomes; |
| Sandhu 2024 | Wrong study design; |
| Brumbaugh 2023 | No intervention / epi descriptive study ; |
| Cui 2023 | No intervention / epi descriptive study ; |
| Arora 2023 | Wrong setting; |
| Torr 2022 | No intervention / epi descriptive study ; |
| Horbar 2020 | No intervention / epi descriptive study ; |
| Brody 2020 | Wrong study design; |
| Kenyon 2012 | Wrong patient population; |
| Feld 2023 | Wrong setting; |
| McBride 2018 | Wrong setting; |

Supplementary File 3: **Preferred Reporting Items for Systematic reviews and Meta-Analyses extension for Scoping Reviews (PRISMA-ScR) Checklist**

| **SECTION** | **ITEM** | **PRISMA-ScR CHECKLIST ITEM** | **REPORTED ON PAGE #** |
| --- | --- | --- | --- |
| **TITLE** | | | |
| Title | 1 | Identify the report as a scoping review. | 1 |
| **ABSTRACT** | | | |
| Structured summary | 2 | Provide a structured summary that includes (as applicable): background, objectives, eligibility criteria, sources of evidence, charting methods, results, and conclusions that relate to the review questions and objectives. | 2 |
| **INTRODUCTION** | | | |
| Rationale | 3 | Describe the rationale for the review in the context of what is already known. Explain why the review questions/objectives lend themselves to a scoping review approach. | 3 |
| Objectives | 4 | Provide an explicit statement of the questions and objectives being addressed with reference to their key elements (e.g., population or participants, concepts, and context) or other relevant key elements used to conceptualize the review questions and/or objectives. | 4 |
| **METHODS** | | | |
| Protocol and registration | 5 | Indicate whether a review protocol exists; state if and where it can be accessed (e.g., a Web address); and if available, provide registration information, including the registration number. | 4 |
| Eligibility criteria | 6 | Specify characteristics of the sources of evidence used as eligibility criteria (e.g., years considered, language, and publication status), and provide a rationale. | 4 |
| Information sources* | 7 | Describe all information sources in the search (e.g., databases with dates of coverage and contact with authors to identify additional sources), as well as the date the most recent search was executed. | 4 |
| Search | 8 | Present the full electronic search strategy for at least 1 database, including any limits used, such that it could be repeated. | Supplement |
| Selection of sources of evidence† | 9 | State the process for selecting sources of evidence (i.e., screening and eligibility) included in the scoping review. | 4 |
| Data charting process‡ | 10 | Describe the methods of charting data from the included sources of evidence (e.g., calibrated forms or forms that have been tested by the team before their use, and whether data charting was done independently or in duplicate) and any processes for obtaining and confirming data from investigators. | 4 |
| Data items | 11 | List and define all variables for which data were sought and any assumptions and simplifications made. | 4 |
| Critical appraisal of individual sources of evidence§ | 12 | If done, provide a rationale for conducting a critical appraisal of included sources of evidence; describe the methods used and how this information was used in any data synthesis (if appropriate). | 5 |
| Synthesis of results | 13 | Describe the methods of handling and summarizing the data that were charted. | 4-5 |
| **RESULTS** | | | |
| Selection of sources of evidence | 14 | Give numbers of sources of evidence screened, assessed for eligibility, and included in the review, with reasons for exclusions at each stage, ideally using a flow diagram. | 5-6 |
| Characteristics of sources of evidence | 15 | For each source of evidence, present characteristics for which data were charted and provide the citations. | 6-7 |
| Critical appraisal within sources of evidence | 16 | If done, present data on critical appraisal of included sources of evidence (see item 12). | 6-7 |
| Results of individual sources of evidence | 17 | For each included source of evidence, present the relevant data that were charted that relate to the review questions and objectives. | 6-7 |
| Synthesis of results | 18 | Summarize and/or present the charting results as they relate to the review questions and objectives. | 6-7 |
| **DISCUSSION** | | | |
| Summary of evidence | 19 | Summarize the main results (including an overview of concepts, themes, and types of evidence available), link to the review questions and objectives, and consider the relevance to key groups. | 7 |
| Limitations | 20 | Discuss the limitations of the scoping review process. | 8-9 |
| Conclusions | 21 | Provide a general interpretation of the results with respect to the review questions and objectives, as well as potential implications and/or next steps. | 9 |
| **FUNDING** | | | |
| Funding | 22 | Describe sources of funding for the included sources of evidence, as well as sources of funding for the scoping review. Describe the role of the funders of the scoping review. | 12 |
